# Assessment of quality of life (QoL) in breast cancer patients by using EORTC QLQ-C30 and BR-23 questionnaires: A tertiary care center survey in the western region of Saudi Arabia

**DOI:** 10.1371/journal.pone.0219093

**Published:** 2019-07-10

**Authors:** Muhammad Imran, Rolina Al-Wassia, Shadi Salem Alkhayyat, Mukhtiar Baig, Bashayer Abdulrahim Al-Saati

**Affiliations:** 1 Department of Surgery, Faculty of Medicine in Rabigh, King Abdulaziz University, Jeddah, Saudi Arabia; 2 Radiation Oncology Unit, King Abdulaziz University, Jeddah, Saudi Arabia; 3 Department of Internal Medicine, Medical Oncology Division, King Abdulaziz University, Jeddah, Saudi Arabia; 4 Department of Clinical Biochemistry, Faculty of Medicine in Rabigh, King Abdulaziz University, Jeddah, Saudi Arabia; 5 Department of Medicine, King Abdulaziz University, Jeddah, Saudi Arabia; University of South Alabama Mitchell Cancer Institute, UNITED STATES

## Abstract

This cross-sectional study is aimed at assessing the quality of life in a cohort of breast cancer patients at the Oncology Department, King Abdulaziz University Hospital (KAUH), King Abdulaziz University (KAU), Jeddah, Saudi Arabia (SA), and to differentiate QoL among different groups. Mean time since diagnosis was 3.97±1.90 years. European Organization for Research and Treatment of Cancer Quality of Life Questionnaires—Core30 and BR23 (EORTC QLQ-C30 & BR23) were used to assess QoL in breast cancer survivors. ANOVA and independent t-test (parametric tests) were used for the categorical variables and Kruskal-Wallis and Mann-Whitney tests used for non-parametric tests. Linear regression analysis was done to measure predictors’ significance and to calculate the coefficient of determination. Two hundred and eighty-four patients completed the survey. Global health status and functional scales, in most of the domains, were high, while symptom scales were moderate-to-low for most items, showing better QoL. Insomnia and fatigue were the most disturbing symptoms. Patients exhibited higher scores for body image and future perspective, while the least score is for sexual functioning. Global health, physical functioning, and role functioning were better in the age group ≤50 years (p<0.05). Premenopausal and perimenopausal patients showed a better level of functioning as compared to postmenopausal patients (p = 0.001). Premenopausal patients scored higher for sexual enjoyment, as compared to peri- and post-menopausal patients (p = 0.04). Systemic therapy side effects were more evident in the breast conservative surgery group. Predictors explained 8% of the variation in Physical functioning (R-squared = 0.08). A predictor that had a remarkable influence on physical functioning, as compared to the other predictors in the model, was menopausal status (P = 0.02). So, it was concluded that the breast cancer patients visiting our institute had a better quality of life regarding overall global health status as well as functional and symptom scales. Some issues, for instance, fatigue, insomnia, hair loss, and others, warrant good supportive therapy.

## Introduction

Breast cancer is the most commonly diagnosed malignancy and the leading cause of death among women worldwide. It is revealed that breast cancer alone is responsible for 30% of newly diagnosed cancer cases in women and there is a probability that one in eight women will develop breast cancer in her lifetime, while 14% of cancer-related deaths are attributed to it [1,2]. It is estimated that a normal woman in the United States carries 12.3% risk of developing breast cancer in her lifetime [1,3]. Breast cancer is common in Arab countries, and although the incidence is lower as compared to the Western population, the number of cases of breast cancer is increasing. Moreover, there are differences between the presentation of breast cancer in European countries and among the Arab population. Women present relatively at a younger age in Arab countries compared to developed nations [4,5], but, on the other hand, they usually present with advanced stages of cancer, or with larger size tumors [6, 7].

Malignancies, especially in advanced stages, are associated with a compromised quality of life (QoL), which can be attributed to physical, psychological and social factors [8]. While comparing cancer survivors with the control population, it is observed that they demonstrate a higher fatigue score and poorer quality of life [9]. Breast cancer is a distinctive entity, as it can severely hamper the physical appearance of affected women, which directly or indirectly affects their QoL, in addition to the fear of cancer, its recurrence, and possible death [10, 11]. Choice of treatment can significantly influence the quality of life in breast cancer survivors. For instance, mastectomy, especially immediate contra-lateral mastectomy, is associated with decreased QoL [12]. This factor has contributed to the establishment of conservative breast surgery, or immediate reconstruction after mastectomy. Either of which is associated with a better quality of life [13]. Depression, sexual problems and fertility-issues can compromise QoL in breast cancer survivors [14]. The economic burden is another factor that contributes to poor QoL [15]. There are specific social issues, especially in developing countries. For instance, separation or divorce from the spouse can compromise QoL in breast cancer survivors [16].

Different tools have been used to assess the quality of life of patients. European Organization for Research and Treatment of Cancer (EORTC) Quality of Life Questionnaires—Core-30 and Breast-23 (QLQ-C30 and QLQ-BR23)—are valid and reliable tools to assess QoL in breast cancer survivors [17–20]. Even the electronic version of patient-related outcome (e-PRO) of EORTC QLQ-C30 is an equally valid and reliable tool [21].

The literature search indicates that there are several factors, which individually or collectively influence the cancer patients’ quality of life (QoL). These contributing factors could be depression, sexual problems and fertility-issues [14], the excessive economic burden [15], social issues such as separation or divorce from the spouse [16], and others. A study from Riyadh region KSA concluded that the patients who had multiple breast tumors or had developed metastasis were experiencing poor QoL [22], while another study also showed poor QoL in breast cancer patients [23].

Keeping in mind the importance of QoL in breast cancer patients and the scarcity of data from Saudi Arabia, especially from the Western region, this study was conducted to assess the quality of life in breast cancer patients by using EORTC QLQ-C30 and BR23 questionnaires in the western region of Saudi Arabia. The present study investigated the quality of life in a cohort of breast cancer patients at the Oncology Department, KAUH, Jeddah, SA, and differentiated QoL among different groups and also explored association of different variables with the domains of QoL. Our study results might help in managing such patients.

## Methods

The Research Ethics Committee of King Abdulaziz University gave the approval for this study, with the research ref. no. 333–15. Written consent was obtained from all the participants on a consent form; the purpose of the study was briefly explained to the participants; and the strict confidentiality of the participants’ information was maintained.

The present survey-based study was carried out in the Oncology Department at KAUH, KAU, Jeddah, SA, and data collection was completed in four months. Being a tertiary care center, a variety of patients with different stages of cancer, age-differences, and modes of presentation, receive treatment here. Management of patients with breast cancer is based on a preset protocol, and each step in the management is evidence-based.

Female breast cancer patients, who were managed at KAUH, with their diagnosis established at least 6 months ago, were eligible to participate in the study. We used the European Organization for Research and Treatment of Cancer Quality of Life QuestionnaireCore-30 and Breast-23 (EORTC QLQ-C30 & -BR23), a tool mostly in use to evaluate QoL in cancer patients, after taking permission from the concerned authorities. Written consent was obtained from all the participants on a consent form, the purpose of the study was briefly explained to the participants and the strict confidentiality of the participants’ information was maintained.

Cohorts of patients being treated at KAUH were asked to fill the questionnaire in Arabic or English language. EORTC QLQ-C30 and -BR23 are validated and reliable tools to assess QoL in patients with breast cancer, and the questionnaires are found to be acceptable in Arab Population as well [17,20,24]. The QLQ-C30 comprises of 30 items categorized to assess different parameters including physical, psychological and social issues, while QLQ-BR23 contains 23 questions to assess important factors in breast cancer survivors. The QLQ-C30 includes global health status, five functional scales, and three symptom scales. There are six single items in it. High scores of functional scales characterize healthy functioning. Similarly, a high score for global health status signifies a higher quality of life. On the other hand, high scores of symptom scales show a high level of problems. Scores for all scales and single items range from 0 to 100. The QLQ-BR23 includes four functional scales and four symptom scales. High scores of functional scales represent better functioning, and high scores of symptom scales show higher issues.

Additional data were also collected regarding age, place of residence, number of family members, history of cancer in the family, marital status, number of kids, employment status, socioeconomic conditions, history of any addictions, and others.

The calculated sample size for this study was 280 using the following formula,

$$n=Z_{1-\alpha /2}{}^{2}p\left(1-p\right)/d^{2}$$

where, n = the minimum sample size, Z_1-α/2 =_ standard normal variate (at 5% type 1 error (*P*<0.05) it is 1.96, p = proportion of breast cancer described by a previous study (27.4%) [5], d = absolute error or precision (5%). The convenience sampling technique was employed to recruit the participants.

Five hundred patients were approached to complete the survey. The researchers of the study, and the data collectors, who were undergraduate medical students, approached the patients. All the patients of breast cancer, visiting the Oncology Department, were contacted in the outpatient department (OPD). Three hundred and ten patients agreed to participate in the research, while 190 patients declined the request. The patients who agreed to participate were given the option to fill the questionnaires in the waiting area. There was a separate comfortable room, used for counselling patients, so the patients sat there at ease and completed the survey. A female staff member, either the female research participant or the data collector, who was briefed about the research questions, accompanied the patients to help clarify any confusing item.

Several types of therapies were given to patients including surgery, chemotherapy, hormone therapy, and radiotherapy. As mentioned, the main purpose of the study was to evaluate the QoL of patients irrespective of the management offered to them. However, certain parameters were taken into consideration to compare the QoL and patients were divided into groups for such variables to compare the results. Variables included: age ≤ or > 50 years; cancer staging—patients with stage 0, 1 and 2 were included in group A, while patients with stages 3 and 4 were included in group B; type of surgery—conservative breast surgery or mastectomy; menopausal status—pre-, peri- and post-menopausal women. The patients were separated according to menopausal status, because it is likely that premenopausal females, being young and dynamic, may have more hopes and passion for fighting the disease and therefore, their QoL is less compromised because of their motivation to cure the problem.

### Statistical analysis

The data were coded and analysed on SPSS 21. We calculated the relevant descriptive statistics for both the questionnaire items. Patients were divided into two groups according to their scores; the patients who scored ≤33 for the functional scales and the global QoL were considered problematic, while the patients who scored ≥66 were considered in good condition. For symptom scales, the score is reversed, i.e., the patients who scored ≤33 were considered in good condition and the patients who scored ≥66 were considered problematic [25,26]. The scores obtained in each domain were the dependent variable in the study while the age, menopausal status, cancer staging, and types of surgery were the independent variables. The ANOVA and independent t-test (parametric tests) were used for the categorical variables and Kruskal-Wallis and Mann-Whitney tests used for non-parametric tests. The Tukey’s test was used for post hoc analysis for finding the differences between groups.

The linear regression analysis was done to measure predictors’ significance and to calculate the coefficient of determination. The dependent variables were global health, physical, emotional, cognitive and social functioning scores, while age, menopausal status, cancer staging, and types of surgery were the independent variable, and were labeled into “Yes” and “No” groups and considered as the model’s predictors. The value of R squared was calculated, and p≤0.05 was taken as significant where the comparison was conducted.

## Results

Three hundred and ten patients completed the survey questionnaire. Twenty-six questionnaires were excluded from the study due to multiple errors. Two hundred and eighty-four patients were included in the study. Their mean and median ages were 51.74±11.59 and 52 years respectively and the mean time since diagnosis was 3.97±1.90 years. Distribution of patients according to their age, cancer-stage, type of surgery, and menopausal status are shown in Table 1, while assessment of the quality of life using EORTC QLQ-C30 and QLQ-BR23 questionnaire is mentioned in Table 2.

**Table 1:** Distribution of the participants according to the cancer staging, mode of surgery and menopausal status.

Variable	N (%)
**Age**	
≤50 years	134(47.2)
>50 years	150(52.8)
**Cancer staging**	
Stage A = 0,1,2	104 (36.6)
Stage B = 3,4	180 (63.4)
**Type of Surgery**	
Conservative breast surgery	112 (39.43)
Mastectomy/modified mastectomy	114 (40.14)
Missing data	58 (20.42)
**Premenopausal**	88(31)
Stage A = 0,1,2	30 (34)
Stage B = 3,4	58 (66)
Conservative breast surgery	38 (43)
Mastectomy/modified mastectomy	34 (39)
Missing data	16 (18)
**Perimenopausal**	96 (34)
Stage A = 0,1,2	30 (31)
Stage B = 3,4	66 (69)
Conservative breast surgery	36 (37)
Mastectomy/modified mastectomy	42 (44)
Missing data	18 (19)
**Postmenopausal**	100 (35)
Stage A = 0,1,2	44 (44)
Stage B = 3,4	56 (56)
Conservative breast surgery	37 (37)
Mastectomy/modified mastectomy	39 (39)
Missing data	24 (24)

**Table 2:** Assessment of quality of life in breast cancer survivors by using EORTC QLQ-C30 and QLQ-BR-23 questionnaires.

Scales	N	No. of items	Mean±SD	95% CI	N (%) Scoring<33.3*	N (%) Scoring>66.7*
**QLQ-C30 questionnaire**						
Global health status/QoL	284	2	67.45±20.51	65.06–69.85	15(5.3)	130(45.8)
Functional scales**						
Physical functioning	284	5	63.61±26.85	60.47–66.75	41(14.4)	141(49.6)
Role functioning	284	2	64.02±34.20	60.03–68.02	36(12.7)	125(44)
Emotional functioning	284	4	67.89±31.10	64.26–71.53	38(13.4)	156(54.9
Cognitive functioning	284	2	72.82±26.47	69.73–75.92	15(5.3)	154(54.2)
Social functioning	284	2	79.63±27.15	76.46–82.80	14(4.9)	187(65.8)
Symptom scales***						
Fatigue	284	3	42.50±26.86	38.71–46.29	80(28.2)	55(19.4)
Nausea and vomiting	284	2	23.47±29.53	18.85–27.29	171(60.2)	21(7.4)
Pain	284	2	38.96±28.39	34.51–42.51	98(34.5)	35(12.3)
Dyspnea	284	1	28.87±32.49	25.05–34.43	132(46.5)	24(8.5)
Insomnia	284	1	42.73±40.00	37.08–48.38	89(31.3)	79(27.8)
Appetite loss	284	1	30.25±34.04	25.44–35.06	119(41.9)	39(13.7)
Constipation	284	1	29.69±37.04	24.01–34.79	146(51.4)	46(16.2)
Diarrhea	284	1	15.25±26.17	11.78–19.32	195(68.7)	11(3.9)
Financial difficulties	284	1	17.13±29.31	13.21–22.17	197(69.4)	15(5.3)
**QLQ-BR-23 questionnaire**						
Functional scales**						
Body image	284	4	79.16±22.83	77.01–83.07	12(4.2)	200(70.4)
Sexual functioning	284	2	37.55±29.65	33.08–41.82	104(36.6)	38(13.4)
Sexual enjoyment	284	2	77.94±27.04	74.12–81.78	7(2.5)	101(35.6)
Future perspective	284	1	67.84±37.05	61.76–72.59	38(13.4)	142(50)
Symptom scales / items***						
Systemic therapy side effects	284	7	42.08±22.28	38.67–45.71	92(42.4)	34(12)
Breast symptoms	284	4	28.34±26.86	24.63–31.39	172(60.6)	27(9.5)
Arm symptoms	284	3	38.18±29.61	33.01–42.11	116(40.8)	47(16.5)
Upset by hair loss		1	45.89±39.66	39.91–50.87	91(32)	76(26.8)

*For functional scales, subjects scoring < 33.3% have problems; those scoring ≥66.7% have good functioning. For symptom scales/symptoms, subjects scoring< 33.3% have good functioning; those scoring = 66.7% have problems.

**For functional scales, higher scores indicate better functioning.

***For symptom scales, higher scores indicate worse functioning

Overall, for EORTC-C30, global health status was high; functional scale in most of the domains especially social functioning and cognitive functioning were high; while, symptom scales were moderate-to-low for most items. The higher scores in global health status showed better QoL. Similarly, higher scores in functional scales indicate better QoL. On the other hand, higher scores in symptom scales show worse QoL [26]. Most of the patients scored >66 (on a scale of 0–100) for global health status and functional scales and this finding is more evident for social functioning, where 65.8% of patients scored >66. Insomnia and fatigue were the most disturbing symptoms followed by pain and loss of appetite. Among symptoms, insomnia was the most distinct and problematic, as 27.8% of patients scored >66. Diarrhea and financial difficulties were the least disturbing symptoms. For QLQ-BR23, patients exhibited higher scores for body image and future perspective, while the least score was for sexual functioning. Regarding symptom scales, hair loss and systemic therapy’s side effects were more disturbing, followed by arm symptoms and breast symptoms. Seventy-six patients scored >66 for the symptom ‘upset by hair loss’ (Table 2).

Quality of life was compared according to different parameters in QLQ-C30 (Table 3). Global health (p = 0.04), physical functioning (p = 0.002), and role functioning (p = 0.01) were better in the age group ≤50 years. Although the score was higher in that age group in other parameters as well, those were not statistically significant. While comparing groups according to menopausal status, physical functioning was found statistically significant—perimenopausal and premenopausal patients showed a better level of functioning as compared to postmenopausal patients (p = 0.001). Staging and type of surgery did not significantly affect QoL (Table 3).

**Table 3:** Comparison of variables in global health and functional scales in QLQ-C30 (N = 284).

Variables	Global health /QoL (QL2) Mean (SD)	Physical functioning Mean (SD)	Role functioning Mean (SD)	Emotional functioning Mean (SD)	Cognitive functioning Mean (SD)	Social functioning Mean (SD)
**Age**						
≤50 yrs (N = 134)	70.02(19.68)	68.85(24.11)	69.40(29.84)	69.34(30.76)	75.62(27.08)	81.84(23.25)
>50 yrs (N = 150)	65.16(21.02)	58.93(28.35)	59.22(37.11)	66.61(31.45)	70.33(25.75)	77.66(30.15)
P-value	0.04	0.002	0.01	0.46	0.09	0.19
**Menopausal status**						
Premenopausal **(N = 88)**	66.57**(**19.26)	69.01**(**24.78)	66.09**(**30.37)	66.38**(**32.71)	72.91**(**27.83)	77.27**(**25.16)
Perimenupausal **(N = 96)**	71.35**(**19.86)	66.94**(**25.51)	68.75**(**33.00)	72.13**(**27.27)	75.34**(**26.15)	83.33**(**25.47)
Postmenopausal **(N = 100)**	64.50**(**21.76)	55.66**(**28.22)	57.66**(**37.71)	65.16**(**32.93)	70.33**(**25.57)	78.16**(**30.12)
P-value	0.06	0.001	0.06	0.25	0.41	0.25
**Cancer staging**						
Stage A = 0,1,2 **(N = 104)**	65.68**(**13.88)	61.98**±**26.84	64.58**±**33.94	66.02**±**31.00	75.00**±**26.58	80.92**±**24.62
Stage B = 3,4 **(N = 180)**	65.38**±**15.19	64.55**±**26.59	63.70**±**34.43	68.98**±**31.20	71.57**±**26.40	78.88**±**28.56
P-value	0.87	0.43	0.83	0.44	0.29	0.54
**Type of Surgery**						
Conservative breast surgery **(N = 111)**	65.69**±**21.83	60.42 **±**25.90	60.81**±**33.15	66.29 **±**31.41	71.32**±**28.44	78.67**±**27.63
Mastectomy/modified mastectomy **(N = 116)**	67.39**±**20.75	65.21**±**27.65	65.65**±**35.34	69.71**±**30.65	72.17**±**25.43	80.14**±**27.29
P-value	.54	.18	.29	.40	.81	.68

Comparison of different variables in QLQ-BR-23 is shown in Table 4. No significant differences were noted for most of the parameters. However, premenopausal patients scored higher, for sexual enjoyment, as compared to peri- and post-menopausal patients (p = 0.04) (Table 4). Patients who underwent conservative breast surgery showed more systematic therapy side effects as compared to the group who underwent the mastectomy.

**Table 4:** Comparison of variables in functional and symptom scales in QLQ-BR23 (N = 284).

Variables	*Functional scales in BR23				**Symptoms scales in BR23			
	Body image	Sexual functioning	Sexual enjoyment	Future Perspective	Systemic therapy side effects	Breast symptoms	Arm symptoms	Upset by hair loss
**Age**								
≤50 yrs (N = 134)	80.65(21.27)	38.18(29.81)	76.14(25.57)	68.90(35.20)	41.64(22.19)	27.05(26.24)	35.15(28.90)	44.02(39.75)
>50 yrs (N = 150)	77.83(24.13)	37.00(29.59)	79.66(28.38)	66.88(38.71)	42.47(22.42)	29.50(27.45)	40.88(30.06)	47.55(39.63)
P-value	0.29	0.73	0.36	0.64	0.75	0.44	0.10	0.45
**Menopausal status**								
Premenopausal **(N = 88)**	81.81**(**19.18)	34.84**(**28.77)	84.28**(**25.83)	67.80**(**34.44)	44.15**(**21.63)	28.97**(**26.80)	35.73**(**28.66)	45.45**(**41.42)
Perimenupausal **(N = 96)**	75.95**(**25.90)	38.88**(**28.47)	75.70**(**24.62)	65.97**(**40.74)	42.26**(**21.94)	29.16**(**27.27)	38.19**(**29.80)	44.09**(**36.99)
Postmenopausal **(N = 100)**	79.91**(**22.50)	38.66**(**31.59)	73.23**(**29.36)	69.66**(**35.79)	40.09**(**23.19)	27.00**(**26.75)	40.33**(**30.36)	48.00**(**40.84)
P-value	0.20	0.58	0.04	0.78	0.45	0.82	0.57	0.78
**Cancer staging**								
Stage A = 0,1,2 **(N = 104)**	79.00**±**24.82	40.38**±**31.04	82.79**±**25.42	68.26**±**36.97	40.97**±**23.54	26.52**±**27.35	35.68**±**29.60	44.23**±**40.07
Stage B = 3,4 **(N = 184)**	79.25**±**21.67	35.92**±**28.77	75.68**±**27.56	67.59**±**37.19	42.72**±**21.55	29.39**±**26.60	39.62**±**29.59	46.85**±**39.50
P-value	0.92	0.22	.08	.88	.52	.38	.28	.59
**Type of Surgery**								
Conservative breast surgery (**N = 111**)	77.02**±**21.88	34.83**±**25.77	78.22**±**27.12	65.76**±**37.18	47.06**±**23.05	29.05**±**26.77	38.03**±**28.80	46.24**±**38.44
Mastectomy/modified mastectomy **(N = 116)**	80.43**±**25.02	40.57**±**31.54	74.89**±**27.12	66.66 **±**38.74	41.07**±**22.39	28.18**±**27.69	40.00**±**29.38	42.60**±**39.62
P-value	.27	.13	.45	.85	.04	.81	.61	.48

*For functional scales, higher scores indicate better functioning.

**For symptom scales, higher scores indicate worse functioning

The predictors explained 8% of the variation in physical functioning (R-squared = 0.08). The predictor that had a remarkable influence on physical functioning as compared to the other predictors in the model was menopausal status (P = 0.02). The same model was built for every domain in QLQ-C30, but no other significant predictors were found in any model (Table 5).

**Table 5:** Linear regression model with parameter estimates for QLQ functional scale.

Variable	Global health /QoL		Physical functioning		Role functioning		Emotional functioning		Cognitive functioning		Social functioning	
	Standardized Coefficient Beta	Significance	Standardized Coefficient Beta	Significance	Standardized Coefficient Beta	Significance	Standardized Coefficient Beta	Significance	Standardized Coefficient Beta	Significance	Standardized Coefficient Beta	Significance
**Constant**	65.67	0.000	61.36	0.000	62.52	0.000	65.82	0.000	75.94	0.000	80.89	0.000
**Age** >50 yrs	-0.054	0.55	-0.054	0.53	-0.107	0.24	0.017	0.85	-0.081	0.37	-0.126	0.17
No = 0												
Yes = 1												
**Postmenopausal**	-0.078	0.40	-0.201	0.02	-0.036	0.69	-0.114	0.21	-0.012	0.90	0.110	.23
No = 0												
Yes = 1												
**Advanced stage cancer**	0.083	0.22	0.120	0.067	0.065	0.33	0.057	0.40	-0.057	.40	-0.274	0.78
No = 0												
Yes = 1												
**Mastectomy/modified mastectomy**	-0.038	0.56	0.088	0.17	0.068	0.30	0.056	0.40	0.015	0.82	.024	0.72
No = 0												
Yes = 1												
R Squared	0.025		0.087		.028		0.019		0.011		0.011	
P-value	0.23		0.00		0.17		0.38		0.64		0.67	

## Discussion

Our study shows that, overall, scores of global health status and functional scales are high, while scores of symptom scales are moderate to low. These scores indicate better QoL in our patients. Insomnia and fatigue are the most distressing symptoms in our study. Pain and appetite loss are other symptoms that affect QoL to a moderate level, while diarrhea and financial difficulties are the least disturbing symptoms (Table 2). This literature supports these findings. In a study from Bahrain with a good sample size (n = 239), global health score was good, and fatigue, sleep disturbance, and pain were the most upsetting symptoms. Moreover, scores for social functioning and emotional functioning were highest and lowest respectively [27]. In a longitudinal study, it was seen that depression, fatigue, and sleep disturbance was expressed as a symptom cluster. So, interventions targeting fatigue might be helpful in combating psychological issues [28]. It was pointed out in a study that breast cancer survivors in younger-age-group experienced fatigue and psychological problems due to the uncertainty associated with their cancer [29]. Even, lower QoL was observed in women with breast cancer symptoms whose diagnoses were still not confirmed, as compared to women in the general population. The psychological domain was prominent in those patients [30]. This factor is again, emphasizing the importance of support for psychological issues. Meisel et al. (2012), in their study on long-term breast cancer survivors, found satisfactory QoL in those patients but there were certain psychological issues [31]. Another study indicated that anxiety disorders have direct effects on compromised QoL in many scales of QLQ-CR30 and QLQ-BR23 including global health status, different social domains, functional and symptom scales [32]. A study from Saudi Arabia showed that emotional function is an important aspect, which is directly related to the patients’ satisfaction among breast cancer patients in palliative care [33]. In a study, fatigue score was higher, showing worse outcome in breast cancer survivors when compared to women without cancer history [15]. A French study described that breast cancer survivors had compromised QoL for different scales, such as fatigue, role functioning, emotional functioning, and physical functioning, and the effect was more significant during the first five years [34].

A positive correlation of perceived social support was found with global health status in a Malaysian study [35]. This factor intimates the importance of social support in breast cancer patients. Our results show the highest scores for social functioning and the lowest for physical functioning. This might signify the social support for our patients in the family and society.

In our study, the functional scale of QLQ-BR23 shows higher scores and better QoL for body image and future perspective. Sexual functioning shows the least score with worse QoL. Hair loss, systemic therapy side effects, arm symptoms, and breast symptoms were the most disturbing among our study group, and these results were compatible with other studies. A study found that hair loss was the leading side effect that affected the quality of life [36]. Jassim & Whitford (2013) showed the highest scores for body image and the lowest for sexual functioning, whereas, the symptom scores were highest for hair loss followed by arm symptoms [27]. Moreover, in their study, regarding QLQ-BR-23, patients with metastasis experienced more systemic therapy side effects, breast symptoms, and arm symptoms, while body image score was significantly poor in patients who underwent a mastectomy [27]. In our study, patients ≤50 years exhibited better QoL in most of the parameters, with significant differences in global health, physical functioning, and role functioning. Similarly, pre- and peri-menopausal women showed better scores in global health scale and most of the functional scales, with a significant difference in physical functioning (Table 3). This is contrary to a Malaysian study, in which it was determined that patients of older-age-group had better QoL [37]. In a systematic review, it was observed that breast cancer survivors of younger-age-group had different problems contributing to compromised QoL, which might include psychological issues and depression; the problem of weight gain; lack of physical activity during treatment; and anxieties about their menopause-related issues [14].

We could not elicit a significant difference among different groups in most of the parameters of QLQ-BR-23. Premenopausal women exhibited better scores, in sexual enjoyment than their counterparts—peri- and post-menopausal women. Another significant finding is the higher score for systematic therapy side effects in the group with conservative breast surgery (Table 4). No significant difference was observed for body image in both groups—whether patients go thorough conservative breast surgery or mastectomy/modified radical mastectomy. The finding cannot be explained with certainty. Perhaps, there might be some social factors involved in it, or there might be issues with the understanding of questions by the patients. Due to the specific dress code and social values of our society, females are not very much conscious about their body figure in public. The factors mentioned above along with family support might be the reason for lesser comprehension by the patients who underwent a mastectomy. This finding is different from other studies. A German study indicated that patients with breast conservative therapy (BCT) showed better QoL in most of the scales of QLQ-C30 and BR-23; some were evident earlier while others showed benefit in the long run [38]. In our study, although, patients of younger age group showed a higher score for body image, this finding is not statistically significant. In a study, it was noted that young patients with breast cancer were concerned more about their bodily appearance [12]. Moreover, it was observed that breast reconstruction showed better QoL and the results were comparable with conservative breast surgery [39].

Specific interpretation can be drawn from our study. Overall, QoL is better among our patients and the findings are compatible with other studies. QoL in breast cancer survivors is compromised in different domains and these areas need attention. Patients of different age groups may elicit different problems associated with that age group. In a study, it was emphasized that quality of life is not static, and patients have altered QoL at different disease stages, for instance, after diagnosis, before and after treatment, and long-term effects of cancer and treatment modalities [40]. It was observed that, if women were provided with social support after their treatment for breast cancer, it could help to decrease mortality and recurrence of cancer, especially during the early post-treatment phase [41]. Some treatment choices may have a positive impact on QoL. A study showed, contrary to common belief, significantly better QoL while using extended adjuvant endocrine therapy in breast cancer patients. It has been suggested to use specific QoL scales instead of the global domain while assessing the effect of treatment on QoL [42].

Our study had limitations. This study was conducted to evaluate overall QoL in breast cancer survivors, so, a few specific issues were not evaluated in detail. Being a questionnaire study, a detailed discussion could not be possible with patients regarding specific matters. Many patients did not respond to the questions about educational status and income, so the comparison was not possible among patients according to their educational level and income.

## Conclusion

The breast cancer patients who visited our institute had a better quality of life regarding overall global health status as well as functional and symptom scales. Patients scored highest in social functioning and lowest in physical functioning. Insomnia and fatigue were the most disturbing symptoms. Similarly, patients scored better in functional scale (QLQ-BR-23), body image and future perspective. Hair loss and systemic therapy side effects were the most disturbing symptoms. Patients of younger-age-group showed better QoL. Some issues, for instance, fatigue, insomnia, hair loss, etc., warrant good supportive therapy to reduce the concerns of patients and to give them psychological support. Future studies can be performed keeping in view specific problems in detail.

## Supporting information

S1 File (SAV)
